# Malaria in Assam: A Challenge

**DOI:** 10.4103/0970-0218.51225

**Published:** 2009-04

**Authors:** Maninder Pal Singh Pardal, Rajvir Bhalwar, Vijay Kumar Mehta, Ajay Mahendraker, Ashwini Kumar Mehta

**Affiliations:** Central Government, Indian Army, Headquarter 21 Mountain Divison - 908 421, C/O 99 APO, India; 1 Department of PSM, AFMC, Sholpaur Road, Pune - 411 040, India; 2 Central Government, Indian Army, Headquarter 16 CORPS, C/O 56 APO, India; 3 Central Government, Indian Army, Military Hospital, Ahmednagar, Maharashtra, India; 4 Central Government, Indian Army, Military Hospital, Saugor, Madhya Pradesh, India

**Keywords:** Assam, malaria, *P falciparum*, *P vivax*

## Abstract

**Research Question:**

What is the trend of malaria and proportion of Plasmodium falciparum infections amongst troops of the Army units deployed in Assam over the last 5 years?

**Study Design:**

Retrospective cross -sectional descriptive study. Setting: Army units deployed in the state of Assam over the last 5 years.

**Participants:**

Population of army units deployed in the state of Assam over the last 5 years.

**Statistical Analysis:**

Percentage, Chi square.

**Results:**

Malaria contributed to 3.60% to 7% of all hospital admissions over the period of 5 years. The overall malaria incidence showed a significantly increasing trend during the study period. (Chi square for linear trend = 5.19; *P* = 0.023). Out of these, *P falciparum* contributed 86% to 98%. The proportion of *P falciparum* infections showed a significantly increasing trend from 2005 to 2006. (Yate's corrected Chi square = 7.123; *P* = 0.008).

## Introduction

Malaria is endemic in most North Eastern States of India with Plasmodium falciparum being the predominant parasite.(1–3) Enhanced morbidity takes a heavy toll on human life because disease outbreaks are an annual event.(1,4,5)

This article attempts to retrospectively review the malaria-attributable morbidity and mortality amongst troops of Army units deployed in Assam over the past 5 years.

## Materials and Methods

### General settings

Flood prone rivers and valleys and interspersing hill ranges constitute the topography of the state of Assam. Difficult terrain and an evergreen rain forest cover nearly 40% of the geographical area and poor communication are the other highlights of this state. Tea plantations and paddy cultivation are the main cash crops. Rainfall is characterized by pre monsoon showers in March and April and heavy rainfall of 2 meters or more during monsoon from July to September.

Relative humidity varying from 70% to 85% throughout the year makes the overall environment conducive for mosquito proliferation, survival, and longevity and also favors active malaria transmission. The movements of Army units in these malaria-infested areas predisposes the personnel to the risk of acquiring malaria infection.

### Research Design

The study design was a retrospective cohort study design.

### Data Collection

Data on morbidity and mortality attributable to malaria for the past 5 consecutive years was collected from the Army units deployed in Assam. In most cases, these units were located at a distance of 4 to 5 hrs from the nearest Military Hospital/Base Hospital. Most of these units have one Regimental Medical Officer. The data we present here is of about 20 army units that were deployed in the state of Assam during the period of study. The data was collected directly from the respective units and also cross checked with data from the Base Hospitals. The same was taken into account to measure morbidity and mortality attributable to malaria.

## Results

Data on morbidity attributable to malaria and the relative proportion of P falciparum and *P vivax* infections over the past 5 consecutive years is presented in Table 1. Out of these, 86% to 98% were *P falciparum* infections; the remaining was *P vivax* malaria. Overall malaria incidence shows a significantly increasing trend during the period of study. (Chi square for linear trend = 5.19; *P* = 0.023). The proportion of P falciparum infections showed a significantly increasing trend from 2005 to 2006. (Yate's corrected Chi square = 7.123; *P* = 0.008).

**Table 1:** Malaria-attributable morbidity and relative proportion of P falciparum and P vivax infections among army units deployed in Assam

Year	No. of malaria cases	Pf	Pv	Percent of Pf cases	Population	Incidence (per 100)
2002	47	44	03	93.62	17803	2.64
2003	85	80	05	94.12	18272	4.65
2004	82	78	04	95.12	18268	4.49
2005	59	51	08	86.44	16386	3.60
2006	85	84	01	98.82	17426	4.88

Over the 5-year period, it was observed that 3.60% to 7% of all hospital admissions were attributable to malaria. Disease burden due to malaria as a proportion of all hospital admissions also generally shows an increasing trend, though not significant [Table 2]. Of the *P falciparum* infections, 3% to 5% developed complicated malaria necessitating a transfer to the Command Hospital. There was no mortality due to malaria during the period of study.

**Table 2:** Relative disease burden attributable to malaria among army units deployed in Assam

Year	Total hospital admissions (all causes)	No. of admissions due to malaria	Disease burden due to malaria (%)
2002	1307	47	3.60
2003	1214	85	7.00
2004	1419	82	5.78
2005	1405	59	4.20
2006	1593	85	5.34

## Discussion

Currently, 2.5 to 3 million cases of malaria occur in India annually as per the available data. Most areas in Assam are considered high risk for acquiring malaria. There is a heavy parasite load in the local population in most parts of the state.(1)

The data presented here is strongly suggestive of the trend of malaria among army units deployed in Assam. It is quite probable that few cases would have been missed due to non reporting. A few possible reasons for the increase in the incidence of malaria among army personnel during the period of study are discussed below:

- Over the years, case detection has increased because of better detection facilities in the form of paracheck kits (Rapid Detection Kit for *P falciparum* and *P Vivax* malaria infections) that have been provided to all Regimental Medical Officers.
- The possibility of an increase in the mosquito population cannot be ruled out, however, that needs to be validated by carrying out studies on vector bionomics.
- Over the years, troops are being exposed to more mosquito bites because of the intensified counter insurgency operations in the thick jungles of Assam, which are heavily infested with mosquitoes.
- Another possible reason for the increase in incidence over the years could be the corresponding increase in incidence in the local civil population of Assam. This will, however, need to be validated by obtaining data from the Joint Director of Health Services (Malaria) of the state of Assam and comparing it with the data from this study.

Army personnel form a highly mobile, non immune population. Thus, malaria is often associated with severe clinical presentation. While all attempts are made for primary and secondary prevention of malaria, geograhical isolation, difficult terrain, and poor communication lead to a delay in reaching the Military Hospital or Base Hospital.

*P falciparum* was the predominant parasite (> 60% to 97%) species in the malaria-ridden tea estates of Assam and in Orissa.(1,6–8) This is comparable with the findings of this study.

The study carried out by Breman, *et al.* revealed *P falciparum* percentage to be 4% to 46% of the total, with a mean of 17%, which is much lower than this present study.(9)

A review of the Federal Health Canada databases for the incidence of malaria in Canada, from 1990 through 2002, documents a range from 364 to 1,029 cases per year, with an average of 538 cases per year during the period (or an average of ≈ 1.8 cases per 100,000 population per year).(10) This is much lower than the incidence in this study.

### Limitations

The present study has its limitations in the sense that vector species identification of the mosquito species responsible for transmitting the infection was not carried out, due to a lack of resources.

## Conclusion

This study attempts to highlight the trend of malaria among army units deployed in the state of Assam. Various important topographical, operational, and environmental factors contributing to the high incidence of malaria in this population have been discussed. The study population, though comprising of army units deployed in the state of Assam, is also suggestive of trends in other North Eastern states of the country where the problem is likely to be as severe if not more.

## Recommendations

Based on the findings of the study, the following recommendation is being submitted:

It is recommended that further studies in this field be conducted to understand in depth the reasons for the increase in the incidence of malaria in this area. These studies should also include studies on vector bionomics and studies of similar data pertaining to the civil population of Assam.
